# Time to first cigarette after waking and risk of incident chronic respiratory diseases in males: A prospective national cohort study

**DOI:** 10.18332/tid/224236

**Published:** 2026-07-11

**Authors:** Jianping Liu, KaiWang Cui, Xia Luo, Xiangwen Gong, Yong Liu

**Affiliations:** 1 Jiangxi Province Key Laboratory of Preventive Medicine, School of Public Health, Nanchang University, Nanchang, China; 2 Department of Respiratory and Critical Care Medicine, Ganzhou Key Laboratory of Respiratory Diseases, Ganzhou Institute of Respiratory Diseases, The Fifth People’s Hospital of Ganzhou, Ganzhou, China

**Keywords:** smoking timing, chronic respiratory disease, cohort study

## Abstract

**INTRODUCTION:**

While cumulative smoking exposure is a well-established risk factor of chronic respiratory disease (CRD), a behavioral indicator of nicotine dependence, the time to first cigarette after waking (TTFC) may provide additional prognostic information. However, prospective evidence linking TTFC to incident CRD, particularly in Chinese populations, remains limited. This study aims to investigate the association between TTFC and incident CRD risk in middle-aged and older men.

**METHODS:**

This prospective cohort study included 5198 Chinese men aged ≥45 years without baseline CRD who were enrolled in the China Health and Retirement Longitudinal Study (CHARLS). TTFC was categorized as >60, 31–60, 6–30, and <6 minutes after waking, with never smokers as the reference group. Cox proportional hazards models were used to estimate hazard ratios (HRs) and 95% confidence intervals (CIs) for incident CRD. Laplace regression was applied to evaluate differences in median disease onset time. Joint effects of TTFC and smoking pack-years were also assessed.

**RESULTS:**

During 44105 person-years of follow-up, 856 incident CRD cases occurred (incidence rate: 19.41 per 1000 person-years). Earlier, TTFC was associated with progressively higher CRD risk in a dose-response manner (p for trend=0.025). Compared with never smokers, participants with TTFC <6 minutes had a significantly higher CRD risk (HR=1.31; 95% CI: 1.02–1.66). Laplace regression showed that TTFC <6 minutes was associated with 0.75 years earlier median disease onset (95% CI: -1.45 – -0.06). Joint exposure analysis indicated that participants with both early TTFC and heavy smoking exposure had the highest risk (HR=1.30; 95% CI: 1.10–1.51), with evidence of additive interaction (attributable proportion, AP=0.177; 95% CI: 0.005–0.349).

**CONCLUSIONS:**

Earlier TTFC is independently associated with increased risk and earlier onset of CRD among Chinese middle-aged and older men. TTFC may serve as a simple and potentially informative behavioral marker for identifying smokers with a higher risk of CRD.

## INTRODUCTION

Chronic respiratory diseases (CRDs), including chronic obstructive pulmonary disease, chronic bronchitis, and other chronic airway disorders, represent one of the core chronic conditions threatening public health globally, with prevalence and attributable health burden remaining persistently elevated^1,2^. Recent systematic analyses based on the Global Burden of Disease Study have demonstrated that the burden of CRD continues to increase substantially, particularly in Asian countries where population aging and sustained tobacco exposure contribute to escalating morbidity and mortality^3,4^. These diseases not only lead to progressive decline in pulmonary function and deterioration of quality of life, but also significantly increase patient mortality, impose substantial caregiving burdens on families, and consume considerable healthcare resources, making them a critical factor affecting healthy life expectancy^5,6^. Therefore, identifying potential risk factors and optimizing prevention strategies to reduce disease burden has become an urgent priority.

Tobacco smoking remains the most important preventable risk factor for CRD, accounting for a large proportion of incident cases and disease progression^3,7^. Recent evidence has further confirmed that higher smoking exposure is associated with increased risks of airway obstruction, chronic bronchitis, and dyspnea in a dose-dependent manner^8^. Existing research has predominantly focused on the association between smoking quantity and CRD, confirming that cumulative smoking exposure, typically measured using pack-years, serves as a well-established predictor of respiratory outcomes. Nevertheless, this measure may not fully capture the heterogeneity in nicotine dependence and individual susceptibility to smoking-related harm.

Time to first cigarette after waking (TTFC) has been increasingly recognized as a simple and reliable behavioral marker of nicotine dependence severity^9^. Biomarker-based investigations have demonstrated that smokers with shorter TTFC exhibit significantly higher serum cotinine concentrations independent of daily cigarette consumption, suggesting enhanced internal nicotine exposure and elevated toxic burden^10,11^. Furthermore, earlier TTFC has been associated with adverse metabolic profiles, including unfavorable lipid levels, providing additional evidence for its role as an indicator of increased systemic harm from tobacco exposure^12^.

Emerging evidence from large-scale prospective cohort studies has highlighted the broader clinical implications of smoking timing beyond traditional smoking quantity measures. Recent analyses from the UK Biobank have demonstrated that earlier TTFC is independently associated with significantly increased risks of multiple chronic diseases, including type 2 diabetes, chronic kidney disease, and atrial fibrillation, even after accounting for genetic susceptibility and conventional lifestyle risk factors^13-15^. Moreover, earlier TTFC has been linked to increased cardiovascular disease incidence and all-cause mortality, indicating that nicotine dependence-related smoking behaviors contribute to disease risk not fully reflected by conventional smoking intensity measures^16^.

With respect to respiratory outcomes, recent prospective evidence from the UK Biobank demonstrated that earlier TTFC was significantly associated with increased risk of incident chronic obstructive pulmonary disease, independent of genetic risk factors and cumulative smoking exposure, highlighting TTFC as a novel behavioral marker associated with CRD risk^17^. Additionally, cross-sectional data from a nationally representative Chinese elderly population revealed that shorter TTFC was significantly associated with higher prevalence of CRD, suggesting that nicotine dependence may play an important role in respiratory disease susceptibility^18^. However, prospective cohort evidence examining the association between TTFC and incident CRD in middle-aged and older Chinese populations remains limited, and whether TTFC provides independent predictive value beyond pack-years. Furthermore, no previous study in China has applied Laplace regression to assess the impact of TTFC on the timing of disease onset, providing additional insight into disease progression.

Therefore, this study uniquely contributes to the literature by providing the first prospective evidence from a nationally representative Chinese men cohort, evaluating both the independent association of TTFC beyond pack-years and its association with earlier disease onset. We further explored potential synergistic effects between nicotine dependence and cumulative smoking burden.

## METHODS

### Study population

This investigation represents a secondary analysis utilizing data from the China Health and Retirement Longitudinal Study (CHARLS), a nationally representative longitudinal survey designed to examine the health and socioeconomic dynamics of middle-aged and older Chinese populations aged ≥45 years. Comprehensive descriptions of the CHARLS methodology, sampling procedures, and data collection protocols have been published previously^19^. In brief, CHARLS employed a multistage stratified probability-proportional-to-size (PPS) sampling strategy. The baseline survey, conducted during 2011–2012, enrolled >17000 participants residing in 450 communities and villages spanning 28 provinces nationwide. Following the baseline survey (2011–2012), participants were followed up in four subsequent waves conducted in 2013, 2015, 2018, and 2020. The CHARLS dataset demonstrates exceptional response rates and rigorous quality control measures, achieving international recognition within the academic research community.

For the present analysis, we utilized data from CHARLS baseline through the most recent available follow-up wave (2020). Due to the limited number of participants with complete data, the present study focused on male participants. The analytical sample was derived through the application of predetermined eligibility criteria. Inclusion criteria were: 1) male, 2) age ≥45 years at baseline, and 3) availability of complete data for TTFC. Exclusion criteria were: 1) presence of a CRD at baseline, 2) missing information on covariates, and 3) loss to follow-up.

The CHARLS study obtained ethical approval from the xxx University, with all study procedures conducted in accordance with relevant guidelines and regulations. Written informed consent was secured from all participants.

### Exposure assessment

TTFC was ascertained at baseline through standardized interviews. Participants were queried regarding their morning smoking behavior using the following item: ‘How soon after waking up do you usually start smoking?’. Four response options were provided for current smokers, including <6 minutes, 6–30 minutes, 31–60 minutes, and >60 minutes after waking. Participants who reported never smoking were classified as non-smokers and served as the reference group, consistent with previous TTFC studies^15^. Based on these responses, participants were categorized into five mutually exclusive groups according to their TTFC interval, with shorter intervals indicating greater nicotine dependence severity and more pronounced tobacco addiction.

### Outcome assessment

The primary outcome was incident CRD occurring during the follow-up period. CRD status was assessed through a structured questionnaire administered at each follow-up wave, wherein participants were asked whether they had ever received a physician diagnosis of chronic lung disease. Chronic lung diseases included chronic bronchitis, emphysema, or pulmonary heart disease, but not tumors or cancer. Individuals who provided an affirmative response to this question were classified as having developed CRD, whereas those who reported no such diagnosis were classified as disease-free. Participants who reported no CRD at baseline but subsequently provided a positive response during any follow-up assessment were identified as incident cases.

### Covariates assessment

Potential confounding variables were selected based on prior literature and biological plausibility. All covariates were measured at baseline. Most covariates were obtained from standardized self-reported questionnaires, except body mass index (BMI). Age was categorized as middle-aged adults (aged 45–64 years) versus older adults (aged ≥65 years), consistent with previous studies of aging populations in China^20^. Education level was classified into three levels: illiterate, primary school and lower, and middle school and higher. The residential setting was dichotomized as rural versus urban, while marital status was categorized as married versus other. Alcohol consumption was classified as never drinker, former drinker, or current drinker. Current drinkers were participants who consumed alcohol more than once per month during the past year; former drinkers had consumed alcohol more than once per month previously but not during the past year; and never drinkers reported never drinking or drinking less than once per month throughout their lifetime. BMI (kg/ m^2^) was computed from measured height and weight and categorized into three groups: <18.5, 18.5–24, and >24^18^. Comorbidity burden was quantified using the Chinese Multimorbidity-Weighted Index (CMWI), which has been validated for assessing multimorbidity among Chinese middle-aged and older adults^21^. Cumulative smoking exposure in pack-years was calculated as average cigarettes per day divided by 20 multiplied by total years of smoking, and was categorized into two groups: less than <20 and ≥20 pack-years^13^.

### Statistical analysis

Continuous data were summarized using means and standard deviations or medians and interquartile ranges as appropriate, whereas categorical data were reported as frequencies and percentages. Comparisons among TTFC groups were conducted using one-way analysis of variance for continuous measures, and chi-squared tests for categorical measures.

Cox proportional hazards models were fitted to estimate hazard ratios and 95% confidence intervals for the association between TTFC and incident CRD. Laplace regression models were used to estimate the 50th percentile differences in time to CRD onset across TTFC categories. A hierarchical modeling approach was implemented: Model 1 adjusted for age, education level, and marital status; Model 2 additionally controlled for BMI, alcohol consumption, and CMWI; Model 3 further incorporated pack-years of smoking. The proportional hazards assumption was examined through Schoenfeld residual tests. Multicollinearity was evaluated using variance inflation factors, with values <5 considered acceptable^22^. A p for trend was computed by entering TTFC categories as an ordinal continuous variable in different models.

Stratified analyses were undertaken to assess potential effect modification across subgroups defined by age category, education level, location of residence, marital status, alcohol consumption pattern, body mass index category, CMWI, and pack-years of smoking. Heterogeneity across strata was evaluated through likelihood ratio tests comparing models with and without interaction terms for multiplicative interaction.

Joint effects of TTFC and pack-years of smoking were investigated by constructing combined exposure categories. Specifically, TTFC was categorized into two groups (<1 hour and ≥1 hour), and pack-years of smoking were dichotomized at 20 years; the joint effects were then analyzed based on these combined categories. Additive interaction was quantified using relative excess risk due to interaction (RERI), the attributable proportion (AP), and the synergy index (S)^23^.

In addition, an exploratory Cox regression analysis was conducted among female participants to assess whether the association between TTFC and CRD was consistent across sexes.

All analyses were conducted using Stata version 15.0 SE. A two-sided p<0.05 was regarded as statistically significant.

## RESULTS

### Baseline characteristics

**Figure 1:**
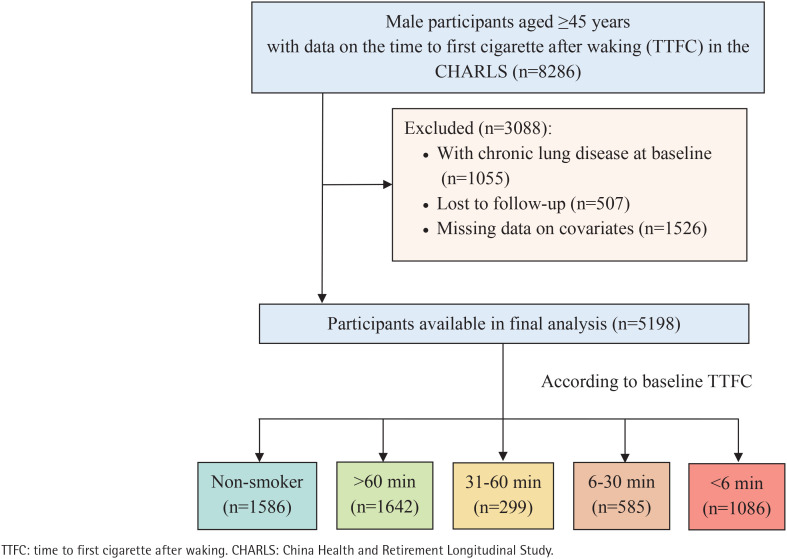
Flowchart of participant selection in the CHARLS cohort study, China, 2011–2020 (N=5198)

A total of 8286 male participants aged ≥45 years with available information on TTFC were initially identified. After excluding individuals with chronic lung disease at baseline (n=1055), those lost to follow-up (n=507), and those with missing covariate data (n=1526), there were 5198 participants included in the final analysis (Figure 1). Participants were categorized into five groups: non-smokers (n=1586), >60 minutes (n=1642), 31–60 minutes (n=299), 6–30 minutes (n=585), and <6 minutes (n=1086).

Baseline characteristics according to TTFC categories are presented in Table 1. Compared with never smokers and those with later TTFC, participants with earlier TTFC were more likely to be middle-aged adults (p<0.001), current alcohol drinkers (p<0.001), living in a village (p=0.049), have higher BMI (p<0.001), and higher pack-years (p<0.001). However, education level (p=0.130), marital status (p=0.170), and CMWI scores (p=0.320) show no significant difference across groups.

**Table 1:** Baseline characteristics of participants by time to first cigarette (TTFC) after waking in the CHARLS cohort study, China (N=5198)

*Characteristics*	*TTFC (minutes)*					*p*
	*Non-smoker* *n (%)*	*>60* *n (%)*	*31–60* *n (%)*	*6–30* *n (%)*	*<6* *n (%)*	
**Total,** n	1586	1642	299	585	1086	
**Age** (years)						<0.001
Middle-aged adults (45–64)	1107 (69.80)	1190 (72.47)	236 (78.93)	474 (81.03)	875 (80.57)	
Older adults (≥65)	479 (30.20)	452 (27.53)	63 (21.07)	111 (18.97)	211 (19.43)	
**Education level**						0.130
Illiterate	191 (12.04)	211 (12.85)	31 (10.37)	70 (11.97)	141 (12.98)	
Primary school and lower	639 (40.29)	682 (41.53)	126 (42.14)	265 (45.30)	489 (45.03)	
Middle school and higher	756 (47.67)	749 (45.62)	142 (47.49)	250 (42.74)	456 (41.99)	
**Residence**						0.049
Rural	921 (58.07)	951 (57.92)	170 (56.86)	327 (55.90)	679 (62.52)	
Urban	665 (41.93)	691 (42.08)	129 (43.14)	258 (44.10)	407 (37.48)	
**Marital status**						0.17
Other	128 (8.07)	161 (9.81)	18 (6.02)	49 (8.38)	100 (9.21)	
Married	1458 (91.93)	1481 (90.19)	281 (93.98)	536 (91.62)	986 (90.79)	
**Alcohol consumption**						<0.001
Never drinker	704 (44.39)	464 (28.26)	95 (31.77)	193 (32.99)	293 (26.98)	
Former drinker	166 (10.47)	198 (12.06)	43 (14.38)	50 (8.55)	107 (9.85)	
Current drinker	716 (45.15)	980 (59.68)	161 (53.85)	342 (58.46)	686 (63.17)	
**BMI** (kg/m^2^)						<0.001
<18.5	76 (4.79)	77 (4.69)	15 (5.02)	40 (6.84)	65 (5.99)	
18.5–24	814 (51.32)	898 (54.69)	179 (59.87)	357 (61.03)	640 (58.93)	
>24	696 (43.88)	667 (40.62)	105 (35.12)	188 (32.14)	381 (35.08)	
**CWMI,** median (IQR)	1.3 (0–2.4)	1.3 (0–2.5)	0.7 (0–2.3)	0.9 (0–2.3)	1.3 (0–2.3)	0.320
Pack-years, median (IQR)	0	16.4 (7.9–27.5)	26.7 (16.7–36.7)	27.5 (19.0–38.3)	33.3 (24.2–48.3)	<0.001

TTFC: time to first cigarette after waking. CHARLS: China Health and Retirement Longitudinal Study. BMI: body mass index. CWMI: Chinese Multimorbidity-Weighted Index. IQR: interquartile range.

### TTFC and incident risk CRD

During a total follow-up of 44105 person-years, 856 incident cases of CRD were documented, corresponding to an incidence rate of 19.41 per 1000 person-years (95% CI: 18.15–20.75) (Supplementary file Table 1).

In Cox proportional hazards models, earlier TTFC was associated with progressively higher risk of CRD. The proportional hazards assumption was satisfied based on Schoenfeld residual tests (p=0.1297), and no evidence of multicollinearity was detected among covariates, with a VIF of 1.22. After full adjustment in Model 3, participants with TTFC less than 6 minutes had a 31% higher risk of developing CRD compared with never smokers (HR=1.31; 95% CI: 1.02–1.66). Similarly, participants with TTFC of 31–60 minutes and 6–30 minutes also exhibited elevated risk estimates, although some associations did not reach statistical significance in fully adjusted models. A significant dose–response relationship was observed across TTFC categories in the fully adjusted model (Model 3) (p for trend=0.025), indicating that shorter TTFC was associated with progressively higher disease risk (Table 2).

**Table 2:** Hazard ratios (HRs), 95% confidence intervals (CIs), and 50th percentile differences (PDs) in years for chronic respiratory disease according to time to first cigarette after waking (TTFC), results from Cox and Laplace regression models in the CHARLS cohort study, China, 2011–2020 (N=5198)

*TTFC (min)*	*n/N (%)*	*HR (95% CI)*			*50th PDs (years) (95% CI)*		
		*Model 1*	*Model 2*	*Model 3*	*Model 1*	*Model 2*	*Model 3*
Non-smoker (ref.)	224/1586 (14.1)	1	1	1	0	0	0
>60	262/1642 (16.0)	1.13 (0.94–1.35)	1.11 (0.93–1.33)	1.09 (0.90–1.33)	-0.35 (-0.87–0.16)	-0.31 (-0.83–0.21)	-0.24 (-0.81–0.32)
31–60	54/299 (18.1)	1.31 (0.98–1.77)	1.31 (0.97–1.77)	1.27 (0.92–1.75)	-0.76 (-1.60–0.08)	-0.75 (-1.59–0.09)	-0.64 (-1.57–0.29)
6–30	105/585 (18.0)	1.29 (1.02–1.63)	1.28 (1.01–1.61)	1.23 (0.94–1.61)	-0.72 (-1.37 – -0.06)	-0.70 (-1.36 – -0.03)	-0.58 (-1.32–0.16)
<6	211/1086 (19.4)	1.37 (1.13–1.65)	1.36 (1.12–1.64)	1.31 (1.02–1.66)	-0.93 (-1.47 – -0.40)	-0.88 (-1.44 – -0.33)	-0.75 (-1.45 – -0.06)
p for trend	856/5198 (16.5)	0.001	0.001	0.025	<0.001	0.001	0.024

Model 1 was adjusted for age, education level, and marital status. Model 2 adjusted as for Model 1 plus BMI, alcohol consumption, and CWMI. Model 3 adjusted as for Model 2 plus pack-years of smoking. HR: hazard ratio. PD: percentile difference. TTFC: time to first cigarette after waking. CHARLS: China Health and Retirement Longitudinal Study.

### TTFC and incident time CRD

To further evaluate the effect of TTFC on disease timing, Laplace regression models were employed to estimate differences in median disease onset time (Table 2).

The median onset of CRD occurred 0.75 years earlier among individuals with TTFC less than 6 minutes (50th percentile difference= -0.75 years, 95% CI: -1.45 – -0.06) after full adjustment. Similar but less pronounced patterns were observed in intermediate TTFC categories. Trend analysis confirmed a significant dose–response relationship between earlier TTFC and earlier disease onset (p for trend=0.024).

### Subgroup analysis

Subgroup analyses were conducted to assess the robustness of the association between TTFC and CRD risk across various population strata (Supplementary file Table 2). The association remained generally consistent across subgroups, with elevated risk estimates observed among participants with shorter TTFC in most strata. For example, compared with non-smokers, participants with TTFC <6 min had a higher risk of CRD among middle-aged adults (HR=1.38; 95% CI: 1.04–1.84), urban residents (HR=1.30; 95% CI: 1.02–1.66), and individuals with BMI >24 kg/ m^2^ (HR=1.80; 95% CI: 1.21–2.70). No statistically significant interactions were observed between TTFC and subgroup variables (all p for interaction >0.05).

### Joint effect of TTFC and smoking pack-years

**Figure 2:**
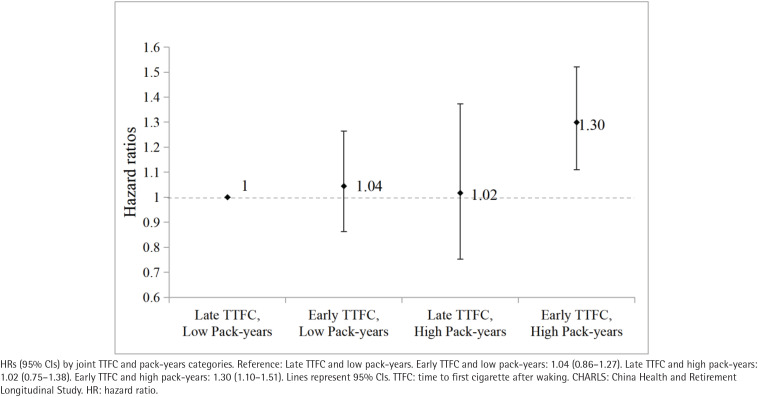
Joint effect of the time to first cigarette after waking (TTFC) and pack-years of smoking on incident chronic respiratory disease in the CHARLS cohort study, China, 2011–2020 (N=5198)

Joint exposure analyses were performed to examine the combined effects of TTFC and smoking pack-years on CRD risk (Figure 2; and Supplementary file Table 3). Compared with participants who had late TTFC and low smoking pack-years, those with both early TTFC and high pack-years demonstrated a significantly increased disease risk, with a 30% higher risk of CRD (HR=1.30; 95% CI: 1.10–1.51, p=0.001). Furthermore, additive interaction analysis revealed evidence of a synergistic effect between early TTFC and high smoking pack-years, with approximately 17.7% of the excess risk attributable to the interaction (AP=0.177; 95% CI: 0.005–0.349). However, the RERI and the synergy index did not reach statistical significance (Supplementary file Table 4).

### Sensitivity analysis

An exploratory analysis among women showed a generally similar pattern of association between TTFC and chronic respiratory disease. Compared with non-smokers, women with TTFC <6 minutes had a higher risk of CRD in model 3 (HR=1.94; 95% CI: 1.09–3.43 in the fully adjusted model; p for trend=0.014) (Supplementary file Table 5).

## DISCUSSION

In this national prospective cohort, we observed that: 1) shorter TTFC was independently associated with increased risk of incident CRD. Compared with non-smokers, participants who smoked within 6 minutes of waking exhibited a 31% higher risk of developing CRD; 2) Laplace regression analysis further showed that early TTFC was associated with an approximately 0.75-year earlier median disease onset; and 3) Joint exposure analyses indicated that the combination of early TTFC and heavy smoking exposure was associated with evidence of additive interaction.

Notably, this study provides the first prospective evidence in a Chinese population demonstrating that TTFC is associated with incident CRD. Previous domestic research on this topic has been limited to cross-sectional designs. Wang et al.^18^ examined a nationally representative elderly cohort and reported that shorter TTFC was associated with higher prevalence of CRD, with particularly pronounced associations observed among female participants, individuals aged ≥90 years, and urban residents^18^. However, the cross-sectional nature of that study precluded determination of temporal sequence and could not exclude the possibility of reverse causation, whereby individuals with respiratory symptoms might alter their smoking patterns. More recently, Zhao et al.^19^ identified associations between shorter TTFC and expiratory airflow limitation using spirometric measurements, yet this investigation similarly lacked longitudinal follow-up. By contrast, our prospective design with extended follow-up establishes the temporal sequence between TTFC assessment and subsequent CRD onset, thereby providing stronger evidence for an association. Furthermore, to our knowledge, this represents the first application of Laplace regression to quantify the impact of TTFC on CRD timing, extending evaluation beyond conventional relative risk estimation to clinically interpretable differences in median onset age. Although the observed advancement in median onset age was approximately 0.75 years, this modest shift may still be meaningful. CRD is progressive, and even a small earlier onset could indicate an accelerated disease trajectory, prolonging the duration of disease burden over the life course. From a population perspective, given the high prevalence of smoking in China, such shifts may have important implications for healthcare utilization and public health planning. By quantifying differences in median onset age, our analysis offers a complementary perspective to relative risk, suggesting that TTFC may be associated not only with CRD risk but also with the timing of disease onset.

Our findings are consistent with accumulating international evidence indicating that smoking timing is an important indicator of health risk independent of conventional smoking quantity metrics. Large prospective analyses from the UK Biobank have demonstrated that shorter TTFC is significantly associated with elevated risks of atrial fibrillation^15^, type 2 diabetes^13^, and chronic kidney disease^14^, even after comprehensive adjustment for smoking intensity, lifestyle factors, and genetic susceptibility. Similarly, shorter TTFC has been linked to increased cardiovascular disease incidence and all-cause mortality^16^, adverse lipid profiles^12^, hypertension in male smokers^24^, and atherosclerosis burden^25^. These multi-system associations underscore the broad health implications of nicotine dependence across organ systems. Biomarker investigations have consistently demonstrated that smokers with shorter TTFC exhibit significantly higher serum cotinine concentrations independent of daily cigarette consumption^10,11^. This finding is particularly important because it suggests that TTFC may reflect an additional dimension of systemic toxic exposure beyond traditional self-reported smoking quantity measures.

With respect to respiratory outcomes specifically, our findings extend and corroborate prior international evidence. Data from the PLCO Cancer Screening Trial demonstrated that shorter time to first morning cigarette was associated with increased COPD risk independent of smoking duration, intensity, and other tobacco-related variables^26^. Selya et al.^27^ reported that TTFC independently predicts pulmonary impairment as measured by FEV1/FVC ratio after controlling for both current and lifetime smoking behavior. A Korean spirometric study found that current smokers with TTFC <30 minutes exhibited significantly greater obstructive airflow limitation compared with those with longer TTFC^28^. Furthermore, shorter TTFC has been established as an independent predictor of lung cancer risk in case-control studies, with a clear dose-response gradient as TTFC decreases^29^. The National Lung Screening Trial similarly reported that tobacco dependence severity, as indexed by TTFC, predicts higher lung cancer incidence, increased mortality, and lower smoking cessation success rates^30^. Most recently, a large UK Biobank prospective study demonstrated that TTFC within 5 minutes of waking significantly increases incident COPD risk, with evidence suggesting potential interaction with genetic susceptibility scores^17^. Our results extend these findings to a Chinese middle-aged and older male population and uniquely demonstrate that early TTFC is associated not only with higher CRD risk but also with earlier disease onset. An exploratory analysis among women revealed a similar direction of association between TTFC and CRD. However, because only a small proportion of women reported smoking and had available TTFC data, these findings should be interpreted cautiously due to limited statistical power and potentially unstable estimates. Further studies with larger samples of female smokers are needed to confirm these findings. Most epidemiological studies have reported positive associations between shorter TTFC and chronic respiratory outcomes. No studies reporting null or inverse associations were identified, although differences in populations, outcomes, and follow-up may affect effect sizes.

Several biological mechanisms may underlie the observed associations between earlier TTFC and CRD risk. TTFC is a well-established marker of nicotine dependence severity^31^. Smokers with shorter TTFC typically exhibit stronger physiological dependence, and adopt more intensive smoking topography, which results in higher systemic absorption of nicotine and tobacco-derived toxicants^10,11,32^. Increased toxic exposure promotes persistent airway inflammation, heightened oxidative stress, epithelial cell injury, and progressive structural remodeling of small airways^33,34^. The stronger association observed among participants with both early TTFC and high cumulative smoking exposure further suggests a joint effect of nicotine dependence and long-term tobacco exposure. Additionally, individuals with shorter TTFC consistently demonstrate lower smoking cessation success rates^30,35^, perpetuating long-term toxic exposure and cumulative airway damage.

### Strengths and limitations

This study possesses several notable strengths alongside certain limitations. The prospective design with longitudinal follow-up enabled temporal assessment of the relationship between TTFC and incident CRD, effectively overcoming the inherent limitations of prior cross-sectional Chinese studies. The application of Laplace regression provided clinically meaningful estimates of differences in disease onset age, representing a novel methodological contribution in this field. However, several limitations warrant consideration. First, CRD was ascertained through self-reported physician diagnosis rather than standardized spirometric confirmation, which may introduce outcome misclassification. In addition, some participants with subclinical or undiagnosed disease may not have been identified during follow-up. Such non-differential misclassification would likely bias the observed hazard ratios toward the null, potentially underestimating the true association between TTFC and chronic respiratory disease risk. Second, only baseline TTFC was used, and potential changes during follow-up were not captured. This lack of time-dependent covariates may have introduced exposure misclassification. However, previous studies indicate that baseline TTFC is relatively stable over time^36^, and is a single-item measure for quantifying nicotine dependence and risk in epidemiological studies^9^. Third, residual confounding from unmeasured environmental or occupational exposures cannot be entirely excluded^3^. Fourth, the exclusively male study population may limit generalizability to female populations. Fifth, exclusion of participants with missing data may have introduced selection bias if the missingness was not completely at random. Sixth, smoking behaviors and several covariates were self-reported, which may be subject to recall bias and social desirability bias. Finally, reverse causation cannot be completely ruled out, as early or subclinical respiratory symptoms may have influenced smoking behaviors, including TTFC.

## CONCLUSIONS

A shorter time to first cigarette after waking is independently associated with an increased risk and earlier onset of CRD among Chinese middle-aged and older men, independent of cumulative smoking exposure. As a simple and cost-free behavioral indicator, TTFC may serve as a simple behavioral marker associated with CRD risk. Future studies incorporating repeated assessments of TTFC and smoking behaviors are needed to confirm the long-term stability of TTFC and to determine whether changes in nicotine dependence over time influence CRD risk.

## Supplementary Material



## Data Availability

The data supporting this research are available from the following sources: http://charls.pku.edu.cn/en
