# Supplementary material for: Time to first cigarette after waking and risk of incident chronic respiratory diseases in males: A prospective national cohort study
Source: Tob Induc Dis. 2026 Jul 11;24:10.18332/tid/224236. doi: 10.18332/tid/224236 (PMC13358854; doi:10.18332/tid/224236)
Supplement: Supplementary file 1 [file TID-24-107-s1.pdf]

 DOI:10.18332/tid/224236

Supplementary Table 1. Incidence of chronic respiratory disease by time to first cigarette after waking (TTFC) in the CHARLS cohort study, China, 2011-2020 (n = 5,198).

TTFC	n/N(%)	Person-years	Incidence rate per 1,000 person-years (95% CI)
Non-smoker	224/1586 (14.1%)	13561	16.51 (14.49, 18.83)
>60 min	262/1642 (16.0%)	13978	18.74 (16.61, 21.16)
31-60 min	54/299 (18.1%)	2502	21.58 (16.53, 28.18)
6-30 min	105/585 (18.0%)	4899	21.43 (17.70, 25.95)
<6 min	211/1086 (19.4%)	9166	23.02 (20.11, 26.35)
Total	856/5198 (16.5%)	44106	19.41 (18.15, 20.75)

Abbreviations: TTFC, time to first cigarette after waking; CHARLS, China Health and Retirement Longitudinal Study; CI, confidence interval.

 DOI:10.18332/tid/224236

Supplementary Table 2. Subgroup analysis of the association between time to first cigarette after waking (TTFC) and chronic respiratory disease in the CHARLS cohort study, China, 2011-2020 (n = 5,198).

Subgroup	TTFC[a]					*P* for interaction
	Non-smoker	>60 min	31-60 min	6-30 min	<5 min	
Age						0.332
middle-aged adults	1 (Reference)	1.29 (1.02, 1.63)	1.43 (0.99, 2.08)	1.43 (1.06, 1.95)	1.38 (1.04, 1.84)	
older adults	1 (Reference)	0.75 (0.52, 1.09)	0.99 (0.50, 1.96)	0.79 (0.43, 1.47)	1.19 (0.94, 1.91)	
Education level						0.322
Illiterate	1 (Reference)	0.8 (0.48, 1.33)	0.79 (0.3, 2.12)	0.93 (0.45, 1.93)	0.85 (0.44, 1.64)	
Primary school and below	1 (Reference)	1.15 (0.86, 1.53)	1.34 (0.83, 2.17)	1.27 (0.85, 1.89)	1.65 (1.17, 2.34)	
Middle school and above	1 (Reference)	1.17 (0.85, 1.6)	1.36 (0.83, 2.24)	1.37 (0.9, 2.09)	1.11 (0.74, 1.66)	
Area of residence						0.325
Village	1 (Reference)	1.04 (0.81, 1.33)	1.02 (0.66, 1.57)	0.99 (0.7, 1.42)	1.13 (0.83, 1.55)	
Urban	1 (Reference)	1.09 (0.9, 1.33)	1.27 (0.92, 1.75)	1.23 (0.94, 1.61)	1.3 (1.02, 1.66)	
Marital status						0.555
Other	1 (Reference)	1.11 (0.9, 1.37)	1.25 (0.89, 1.76)	1.27 (0.96, 1.68)	1.27 (0.98, 1.65)	
Married	1 (Reference)	0.93 (0.49, 1.74)	1.42 (0.49, 4.12)	0.89 (0.36, 2.23)	1.54 (0.74, 3.2)	
Alcohol consumption						0.609
Never drinker	1 (Reference)	1 (0.72, 1.41)	1.49 (0.88, 2.54)	1.55 (1.01, 2.37)	1.41 (0.94, 2.12)	
Former drinker	1	1.12 (0.63,	1.43 (0.63,	1.39 (0.6,	1.72 (0.85,	

	(Reference)	1.98)	3.24)	3.22)	3.51)	
Current drinker	1 (Reference)	1.08 (0.82, 1.41)	1.04 (0.64, 1.67)	0.99 (0.67, 1.45)	1.13 (0.8, 1.59)	
BMI						0.350
<18.5	1 (Reference)	1.1 (0.52, 2.33)	1.71 (0.52, 5.64)	1.03 (0.37, 2.89)	0.66 (0.25, 1.79)	
18.5-24	1 (Reference)	1.01 (0.78, 1.31)	1.02 (0.66, 1.57)	1.11 (0.78, 1.58)	1.09 (0.79, 1.52)	
>24	1 (Reference)	1.2 (0.87, 1.66)	1.52 (0.87, 2.66)	1.38 (0.86, 2.23)	1.8 (1.21, 2.7)	
CWMI						0.826
< 1.3	1 (Reference)	1.05 (0.78, 1.42)	1.03 (0.63, 1.69)	1.12 (0.75, 1.68)	1.25 (0.86, 1.81)	
≥ 1.3	1 (Reference)	1.09 (0.84, 1.42)	1.42 (0.91, 2.19)	1.22 (0.84, 1.77)	1.28 (0.92, 1.78)	
Pack years of smoking						0.149
< 20	1 (Reference)	1.09 (0.88, 1.35)	1.58 (0.98, 2.52)	0.83 (0.51, 1.35)	1.22 (0.82, 1.82)	
≥ 20	1 (Reference)	0.82 (0.64, 1.04)	0.83 (0.57, 1.19)	1.01 (0.78, 1.31)	1.02 (0.83, 1.34)	

[a] Model was adjusted for adjusted for age, education, marital status, BMI, alcohol consumption, CWMI, and pack years of smoking, except for the stratification variable itself.

Abbreviations: TTFC, time to first cigarette after waking; CHARLS, China Health and Retirement Longitudinal Study; BMI, body mass index; CWMI, Chinese Multimorbidity-Weighted Index.

 DOI:10.18332/tid/224236

Supplementary Table 3. Joint effect of time to first cigarette after waking (TTFC) and smoking pack-years on incident chronic respiratory disease in the CHARLS cohort study, China, 2011-2020 (n = 5,198).

**Joint exposure**		N	Chronic respiratory disease		
TTFC	pack years of smoking		n	HR[a] (95% CI)	P
Late	Low	2618	394	Reference	
Early	Low	310	48	1.04 (0.86, 1.27)	0.645
Late	Higher	909	146	1.02 (0.75, 1.38)	0.906
Early	Higher	1361	268	1.30 (1.10, 1.51)	0.001

[a] Adjusted for age, education, marital status, BMI, alcohol consumption, CWMI, and pack years of smoking.

Abbreviations: TTFC, time to first cigarette after waking; CHARLS, China Health and Retirement Longitudinal Study; HR, hazard ratio; CI, confidence interval.

Supplementary Table 4. Estimates and 95% confidence intervals for measures of additive interaction in the CHARLS cohort study, China, 2011–2020 (n = 5,198)

Measure	Estimate	Lower	Upper
Relative excess risk due to interaction (RERI)	0.229	-0.002	0.460
the attributable proportion (AP)	0.177	0.005	0.349
the synergy index (S)	4.568	0.115	182.020

Abbreviations: CHARLS, China Health and Retirement Longitudinal Study; RERI, Relative excess risk due to interaction; AP, the attributable proportion; S, the synergy index.

 DOI:10.18332/tid/224236

Supplementary Table 5. Hazard ratios (HRs), 95% confidence intervals (CIs), and 50th percentile differences (PDs) in years for chronic respiratory disease according to time to first cigarette after waking (TTFC) in male: results from Cox and Laplace regression models in the CHARLS cohort study, China, 2011‑2020 (n = 6852)..

**TTFC**	**n/N(%)**	**HR (95% CI)**		
		Model 1[a]	Model 2[b]	Model 3[c]
Non-smoker	801/6542 (12.2%)	1 (Reference)	1 (Reference)	1 (Reference)
>60 min	32/169 (18.9%)	1.6(1.12, 2.28)	1.49(1.05, 2.13)	1.43(0.97, 2.09)
31-60 min	5/20 (25.0%)	2.29(0.95, 5.53)	2.03(0.84, 4.91)	1.91(0.78, 4.7)
6-30 min	8/38 (21.1%)	1.86(0.93, 3.74)	1.63(0.81, 3.28)	1.47(0.69, 3.13)
<6 min	21/83 (25.3%)	2.24(1.45, 3.46)	2.21(1.43, 3.42)	1.94(1.09, 3.43)
P for trend	867/6852 (12.7%)	0.001	0.001	0.014

[a] Model 1 was adjusted for age, education, and marital status.

[b] Model 2 was adjusted for age, education, marital status, BMI, alcohol consumption, and CWMI.

[c] Model 3 was adjusted for age, education, marital status, BMI, alcohol consumption, CWMI, and pack years of smoking.

Abbreviations: HR, hazard ratio; CI, confidence interval; PD, percentile difference; TTFC, time to first cigarette after waking; CHARLS, China Health and Retirement Longitudinal Study.

© **2026 Liu J. et al.**

The content has been provided by the author(s) and has not been reviewed, verified, or endorsed by European Publishing. It may not have undergone peer review. The views, opinions, and recommendations expressed are solely those of the author(s) and do not necessarily reflect the position of European Publishing. European Publishing accepts no responsibility or liability for any consequences arising from the use of, or reliance on, this content.
